# Supplementary material for: SARS-CoV-2 causes chronic lung inflammation and impaired respiratory capacity in aged Roborovski dwarf hamsters
Source: J Virol. 2025 Aug 11;99(9):e00755-25. doi: 10.1128/jvi.00755-25 (PMC12456015; doi:10.1128/jvi.00755-25)
Supplement: Supplemental material — Tables S1 to S3; Fig. S1 to S3. [file jvi.00755-25-s0003.docx]

**Chronic lung inflammation and impaired respiratory capacity in aged Roborovski dwarf hamsters infected with SARS-CoV-2**

Amirhossein Karimi^1^, Carolin M Lieber^1^, Kaori Sakamoto^2^, Richard K Plemper^1^*

**Supplementary Information**

**Supplementary Tables**

**Supplementary Table 1. Determination of normal range WBP metrics in Roborovski dwarf hamster subgroups of different age and sex**.

| **Parameter** | **Unit** | **5th**  **Percentile** | **95th**  **Percentile** | **5th**  **Percentile** | **95th**  **Percentile** | **5th**  **Percentile** | **95th**  **Percentile** | **5th**  **Percentile** | **95th**  **Percentile** | **5th**  **Percentile** | **95th**  **Percentile** | **5th**  **Percentile** | **95th**  **Percentile** | **5th**  **Percentile** | **95th**  **Percentile** |
| --- | --- | --- | --- | --- | --- | --- | --- | --- | --- | --- | --- | --- | --- | --- | --- |
| **Ti** | ms | 107.5 | 185.2 | 118.1 | 193.9 | 129.6 | 191.6 | 116.6 | 175.7 | 107.6 | 225.9 | 81.52 | 150.2 | 109.1 | 187.5 |
| **Te** | ms | 220.9 | 410.2 | 237.4 | 496 | 282.5 | 487.2 | 247.9 | 442.6 | 213.3 | 544.7 | 129 | 456.6 | 237.9 | 452.1 |
| **PIF** | ml/s | 1.943 | 3.572 | 1.931 | 3.245 | 1.965 | 3.198 | 2.066 | 3.49 | 2.051 | 3.796 | 1.949 | 4.102 | 2.005 | 3.446 |
| **PEF** | ml/s | 1.084 | 2.096 | 1.009 | 2.163 | 1.123 | 1.9 | 1.158 | 2.205 | 0.8983 | 2.643 | 0.9601 | 2.87 | 1.094 | 2.203 |
| **TV** | ml | 0.1355 | 0.2476 | 0.1464 | 0.216 | 0.1537 | 0.2759 | 0.1544 | 0.2669 | 0.1803 | 0.2924 | 0.1312 | 0.2272 | 0.1498 | 0.2578 |
| **RT** | ms | 137.3 | 247.5 | 140.3 | 229.4 | 165 | 235 | 148.4 | 251.3 | 138.2 | 296.8 | 90.53 | 248.2 | 143.5 | 245.4 |
| **MV** | ml/min | 20.31 | 41.7 | 19.31 | 39.72 | 20.28 | 35.78 | 21.84 | 43.52 | 20.82 | 46.96 | 19.41 | 56.21 | 20.99 | 42.79 |
| **F** | bpm | 122.2 | 224.2 | 113.4 | 211.3 | 109.7 | 179.8 | 116.1 | 201.7 | 99.48 | 237.7 | 134.7 | 307.1 | 114.1 | 224.5 |
| **EIP** | ms | 2.375 | 5.802 | 2.066 | 4.34 | 2.069 | 4.585 | 2.144 | 5.627 | 2.161 | 5.216 | 1.525 | 6.607 | 2.151 | 5.197 |
| **EEP** | ms | 25.55 | 51.79 | 28.05 | 76.29 | 34.24 | 66.94 | 30.09 | 62.62 | 23.56 | 86.38 | 12.2 | 81.87 | 28.12 | 66.2 |
| **PenH** | N/A | 0.31 | 0.4991 | 0.3366 | 0.6975 | 0.3355 | 0.5803 | 0.3201 | 0.5706 | 0.2583 | 0.5759 | 0.2279 | 0.4977 | 0.2925 | 0.5621 |
| **EF50** | ml/s | 0.7697 | 1.608 | 0.7141 | 1.594 | 0.7578 | 1.29 | 0.7914 | 1.68 | 0.6809 | 1.905 | 0.6851 | 2.219 | 0.7627 | 1.651 |
| Male (2-6 mo)* | | Female (2-6 mo)** | | Male (7-11 mo)*** | | Female (7-11 mo)**** | | Male (12-16 mo)***** | | Female (12-16 mo)****** | | Universal | |  |  |

*n=26

**n=37

***n=63

****n=55

*****n=26

******n=28

Ti, inspiratory time; Te, expiratory time; PIF, peak inspiratory flow; PEF, peak expiratory flow; TV, tidal volume; RT, relaxation time; MV, minute volume; F, frequency of breathing; EIP, end-inspiratory pause; EEP, end-expiratory pause; PenH, enhanced pause; EF50, 50% of expiratory flow; ms, millisecond; ml/s, milliliter/second; bpm, breaths per minute.

**Supplementary Table 2. Determination of minimum time required for full dwarf hamster recovery from 30 seconds methacholine exposure**.

| **Dwarf hamsters (4-5 months)** | **Methacholine dose (mg/ml)** | **Delivery cycle** | **Exposure time** | **Post exposure WBP** | **Clinical signs** | **Actions taken** |
| --- | --- | --- | --- | --- | --- | --- |
| Males (n=4) | 5 | 15% | 30 seconds | 5 minutes | Increased respiratory rate | N/A |
| Females (n=4) | 5 | 15% | 30 seconds | 5 minutes | Increased respiratory rate | N/A |
| Males (n=4) | 10 | 15% | 30 seconds | 5 minutes | Tachypnea | N/A |
| Females (n=4) | 10 | 15% | 30 seconds | 5 minutes | Tachypnea | N/A |
| Males (n=4) | 20 | 15% | 30 seconds | 5 minutes | Tachypnea, shaking, slightly muddy mucosal membrane | Exposure stopped. Animals exposed to fresh air. Full recovery within 15 minutes |
| Females (n=4) | 20 | 15% | 30 seconds | 5 minutes | Tachypnea, shaking, followed by transient apnea, muddy mucosal membrane | Exposure stopped. Animals exposed to fresh air. Full recovery within 15 minutes |

**Supplementary Table 3.** **Primer sequences of Roborovski dwarf hamster cytokines used for qPCR**.

| **Cytokine** | **INF-γ** | **INF-β** | **TNF-α** | **IL-2** | **IL-6** | **IL-10** | **IL-12** | **IL-17** | **GAPDH** |
| --- | --- | --- | --- | --- | --- | --- | --- | --- | --- |
| **Forward primer sequence** | CTATGTCTGGCTGCTACGGC | CTTCACCTCCATAGGGATCTTG | CCCACACTGTCAGCCGTATT | TGCTTCTGAGCAGGATCAATAA | GTGACTCCAGCTTCTCCATTAG | CTAACTGCACCCACTTCCCA | CAGGCTCTGAATCTCAATGGT | CGCTCGAATGGAAGAGTATGAG | GGTGCCGAGTATGTTGTGGA |
| **Reverse primer sequence** | AGGTCTGCCTTGATGGTGTC | GCTCAAACAAAGCAGCACTAA | AGGTTGACCTCAGCACGAAG | AGGCAGTGGAGATGTTTCAG | CAAGAGGTAAAGGATCCAGGTAAA | TTGGCAACCCAAGTAACCCT | GGATGCTGAAAGCCTGTAGTAG | GGAGACAGGCTTCTCTTGTTAG | AGTGATGGCATGGACTGTGG |

**Supplementary Figures**


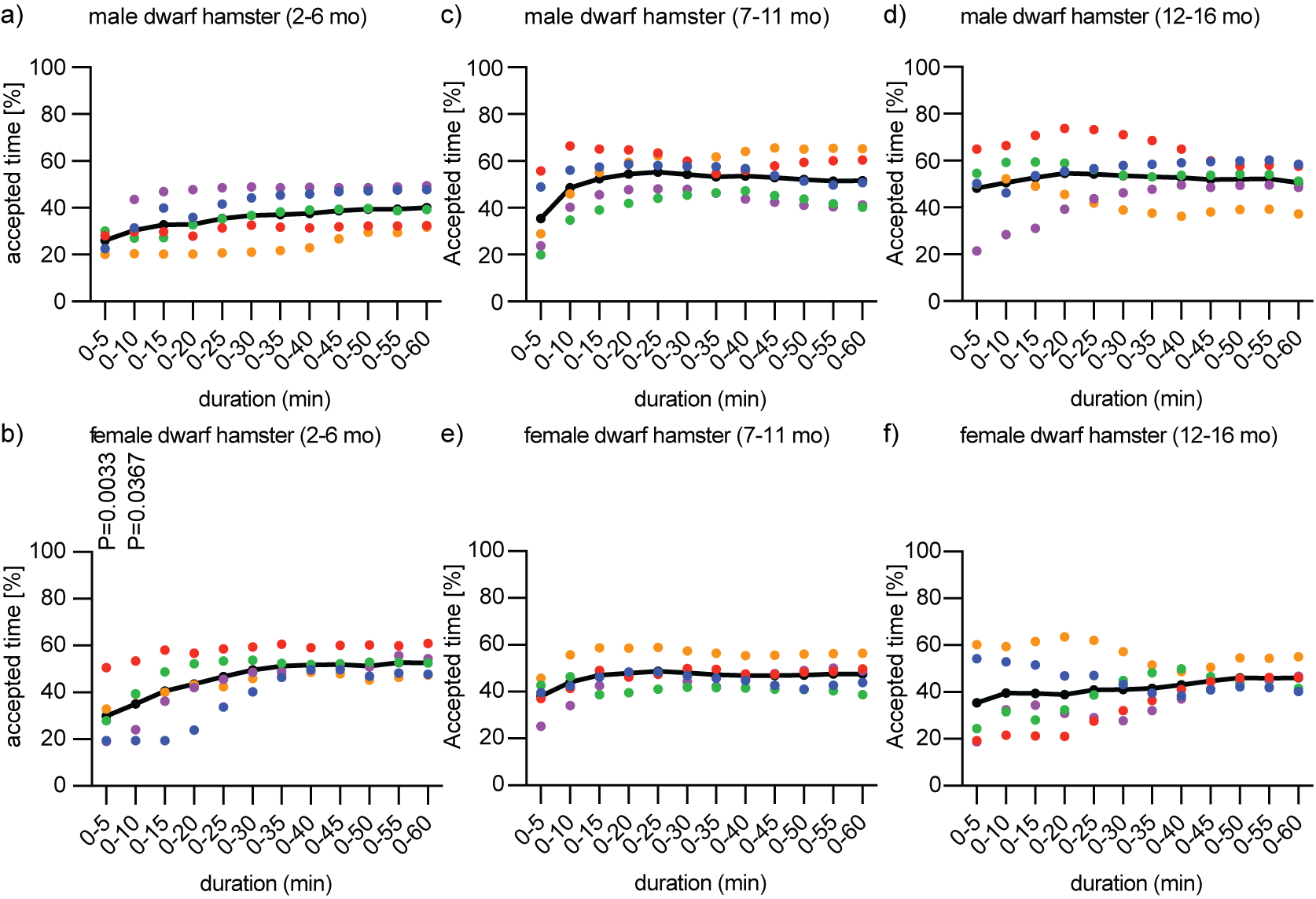
 **Supplementary Figure 1. Percentage of accepted events in different time frames of WBP recording in Roborovski dwarf hamsters**. **a-f)** Uninfected dwarf hamsters of distinct ages and sexes (n=5/group). Each color shows an individual replicate (individual animals), black lines connect sample means. Significance of differences was evaluated by one-way ANOVA with Dunnett’s multiple comparison post-hoc test, comparing the means of each time point to the same group means after 60-minute recording (only P<0.05 are shown).

**
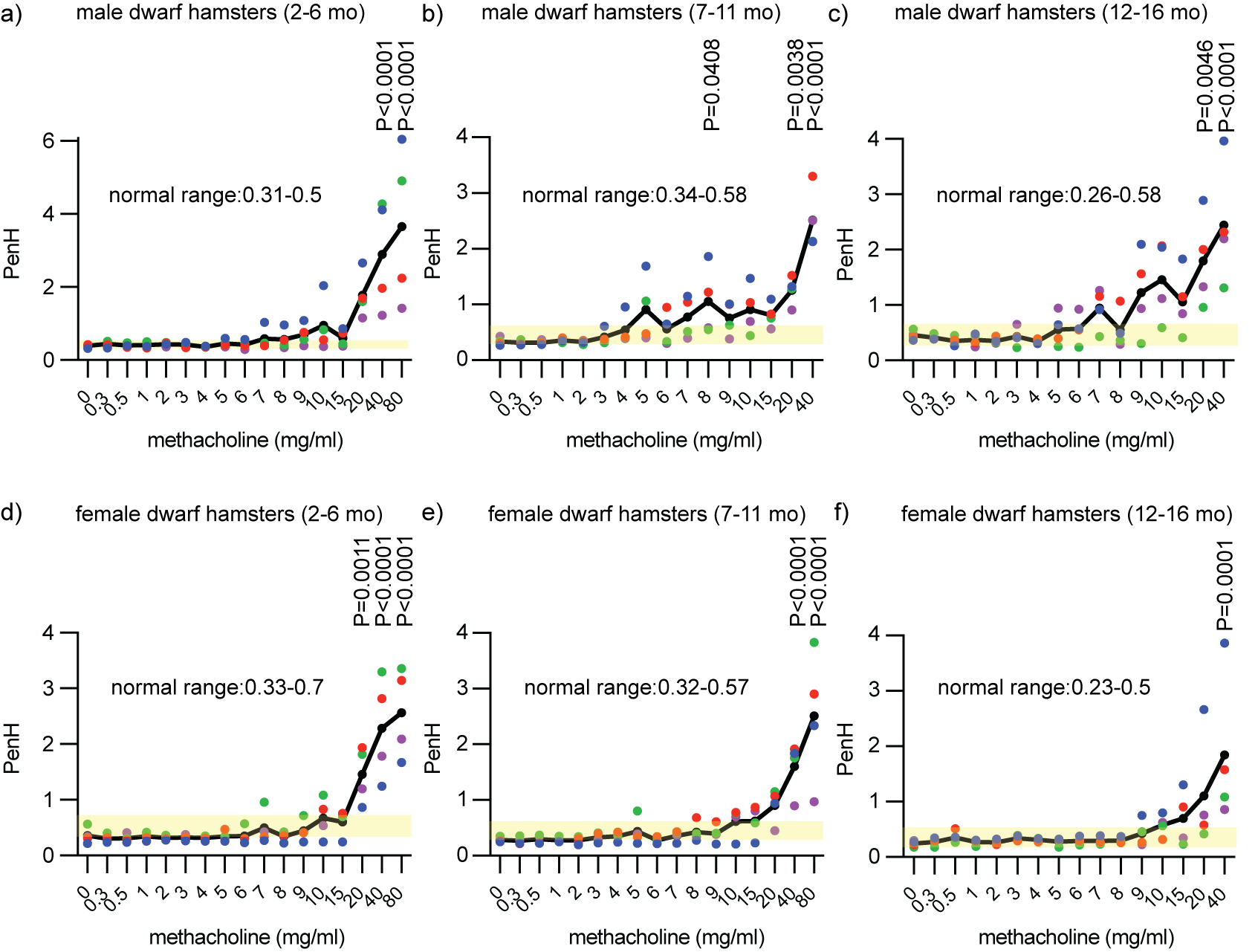
**

**Supplementary Figure 2.** **Methacholine dose-range finding for stress-PenH measurements. a-f)** PenH (mean) comparison after exposure to different dose-level methacholine in the different age and sex groups. Statistical analysis with one-way ANOVA with Dunnett’s multiple comparison post-hoc test (P<0.05 are shown). PenH normal ranges for each age/sex group are given by yellow bars. Biological repeats (independent animals) are shown in different colors (n=4).


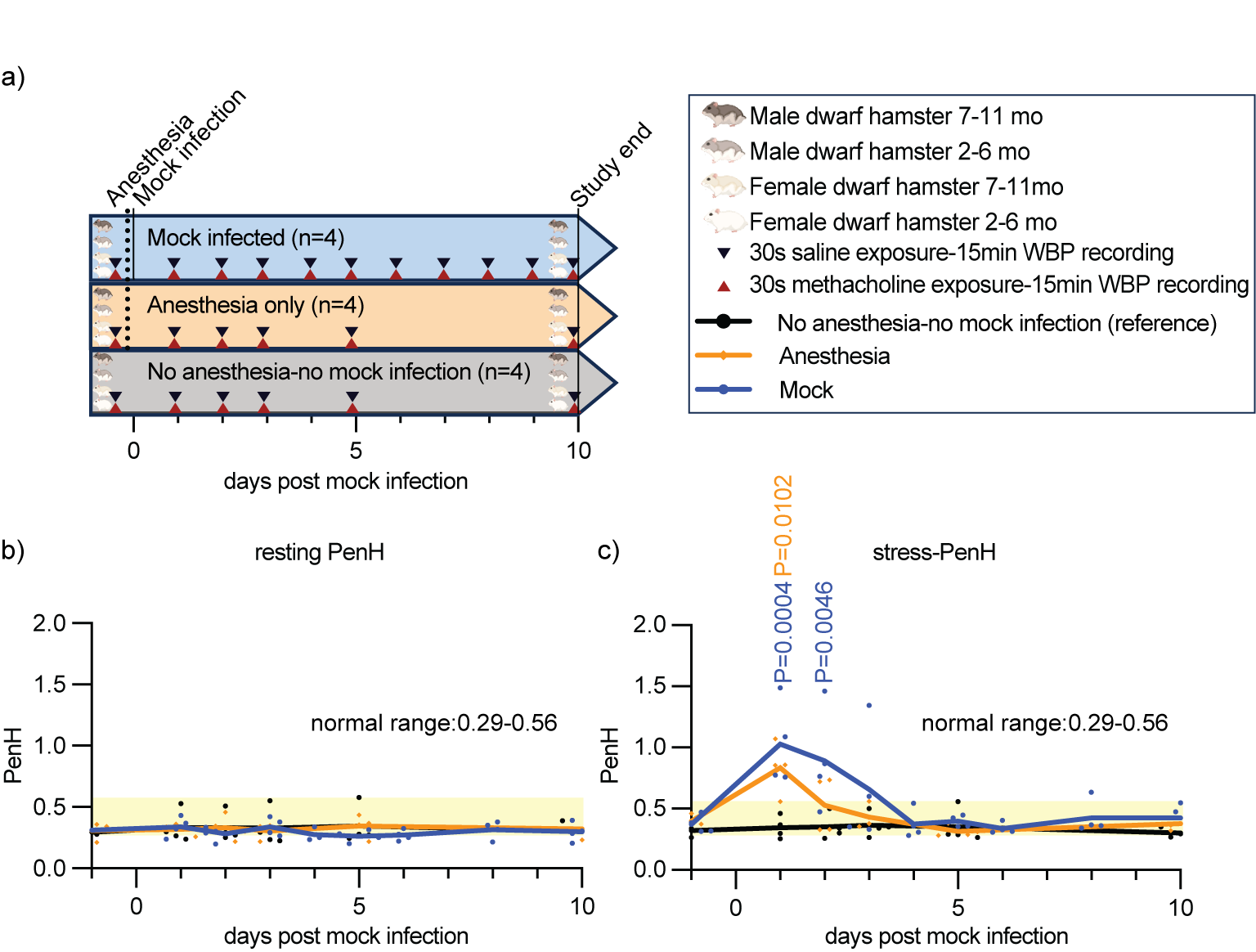


**Supplementary Figure 3. Anesthesia and mock infection transiently increase stress-PenH. a)** Study schematic. Mock infection intranasally with 50 µl PBS. The anesthesia-only group received ketamine + dexmedetomidine (IP). The reference group did not receive anesthesia or intranasal PBS (n=4/group). **b,c)** Saline solution was nebulized to determine the effect of nebulization on PenH in the absence of methacholine (b), 4 mg/ml methacholine was used for stress-PenH (c). Normal ranges are indicated (yellow bars). Statistical significance of differences was evaluated by two-way ANOVA with Dunnett’s multiple comparison post-hoc test (P<0.05 are shown).
